# Editorial: From Raw MEG/EEG to Publication: How to Perform MEG/EEG Group Analysis With Free Academic Software

**DOI:** 10.3389/fnins.2022.854471

**Published:** 2022-04-13

**Authors:** Arnaud Delorme, Robert Oostenveld, Francois Tadel, Alexandre Gramfort, Srikantan Nagarajan, Vladimir Litvak

**Affiliations:** ^1^CerCo, CNRS, Paul Sabatier University, Toulouse, France; ^2^Swartz Center for Computational Neurosciences, Institute of Neural Computation, University of California, San Diego, San Diego, CA, United States; ^3^Donders Institute for Brain, Cognition, and Behaviour, Radboud University, Nijmegen, Netherlands; ^4^NatMEG, Karolinska Institutet, Stockholm, Sweden; ^5^Signal and Image Processing Institute, University of Southern California, Los Angeles, CA, United States; ^6^Université Paris-Saclay, Inria, CEA, Palaiseau, France; ^7^Department of Radiology and Biomedical Imaging, University of California, San Francisco, San Francisco, CA, United States; ^8^Welcome Centre for Human Neuroimaging, UCL Queen Square Institute of Neurology, London, United Kingdom

**Keywords:** EEG, MEG, iEEG, BIDS, pipeline

Free and open-source academic toolboxes have gained increasing prominence in the field of MEG/EEG research to disseminate cutting-edge methods, share best practices between different research groups, and pool resources for developing essential tools for the MEG/EEG community. Large and vibrant research communities have emerged around several of these toolboxes in recent years. Training events are regularly held around the world where the basics of each toolbox are explained by its respective developers and experienced power users. However, most training material and tutorials only show analysis of a single “typical best” subject, whereas most real MEG/EEG studies involve group data analysis. It is then left to the researchers to figure out how to make the transition and obtain group results. This special Research Topic addresses this gap by publishing detailed descriptions of complete group analyses for which code and data are also shared. The level of detail of the description should be such that the readers will be able to fully reproduce the analysis and results and port the analysis to their own data.

A total of 25 articles, summarized in Table 1, were accepted for this special issue. In particular to foster comparable analysis with different tools and strategies, we encouraged authors to reuse a dataset containing responses to face stimuli acquired by Richard Henson and Daniel Wakeman (Wakeman and Henson, 2015; https://openfmri.org/dataset/ds000117/) (HW dataset). This dataset is formatted following the Brain Imaging Data Structure specification (Gorgolewski et al., 2016), which has become increasingly popular in the MEG (Niso et al., 2018), EEG (Pernet et al., 2019) and iEEG fields (Holdgraf et al., 2019). The specific dataset contains multiple modalities, including EEG (with digitized electrode positions), MEG, fMRI, and anatomical MRI, making it suitable for demonstrating multimodal analysis pipelines. Out of the 25 published articles, 10 are using this data (Table 1). All other articles used data that is also publicly available.

**Table 1:** Article part of the special issue by order of publication date.

**Title**	**Authors**	**Script location**	**License**	**Data** **type**	**Primary outcome**	**Language**	**Uses**	**Data**
The Detection of Phase Amplitude Coupling during Sensory Processing	Seymour et al.	Sup. mat.		MEG	Phase amplitude coupling	MATLAB	Fieldtrip	Yes
Group Analysis in MNE-Python of Evoked Responses from a Tactile Stimulation Paradigm: A Pipeline for Reproducibility at Every Step of Processing, Going from Individual Sensor Space Representations to an across-Group Source Space Representation	Andersen	GitHub		MEG	Beamformer	Python	MNE	Yes
Group-Level EEG-Processing Pipeline for Flexible Single Trial-Based Analyses Including Linear Mixed Models	Frömer et al.	OSF		EEG	Linear mixed model	MATLAB and R	EEGLAB; Fieldtrip	Yes
The Harvard Automated Processing Pipeline for Electroencephalography (HAPPE): Standardized Processing Software for Developmental and High-Artifact Data	Gabard-Durnam et al.	HAPPE site	GNU/ GPL	EEG	Automated pre-processing	MATLAB	EEGLAB	Yes
Computational Testing for Automated Preprocessing 2: Practical Demonstration of a System for Scientific Data-Processing Workflow Management for High-Volume EEG	Cowley and Korpela	CTAP site	MIT	EEG	Automated pre-processing	MATLAB	EEGLAB	Yes
Group Analysis in FieldTrip of Time-Frequency Responses: A Pipeline for Reproducibility at Every Step of Processing, Going From Individual Sensor Space Representations to an Across-Group Source Space Representation	Andersen	Personal site		MEG	Beamformer	MATLAB	Fieldtrip	Yes
How to Build a Functional Connectomic Biomarker for Mild Cognitive Impairment From Source Reconstructed MEG Resting-State Activity: The Combination of ROI Representation and Connectivity Estimator Matters	Dimitriadis et al.	Figshare		MEG	Connectivity analysis	MATLAB	Fieldtrip	Yes
Source-Modeling Auditory Processes of EEG Data Using EEGLAB and Brainstorm	Stropahl et al.	Sup. Mat.		EEG	Source analysis	MATLAB	EEGLAB; Brainstorm	Yes
A Student's Guide to Randomization Statistics for Multichannel Event-Related Potentials Using Ragu	Habermann et al.	Ragu site	GNU/ GPL	EEG	ERP; Microstates	MATLAB		Yes
From ERPs to MVPA Using the Amsterdam Decoding and Modeling Toolbox (ADAM)	Fahrenfort et al.	ADAM site	GNU/ GPL	EEG	MVPA	MATLAB	EEGLAB; Fieldtrip	Yes (HW)
Group-Level Multivariate Analysis in EasyEEG Toolbox: Examining the Temporal Dynamics Using Topographic Responses	Yang et al.	EasyEEG site	GNU/ GPL	EEG	ERP; Classification	Python	MNE	Yes (HW)
BEAPP: The Batch Electroencephalography Automated Processing Platform	Levin et al.	BEAP site	GNU/ GPL	EEG	Automated pre-processing	MATLAB	EEGLAB; PREP; HAPPE	Yes ^†^
A Reproducible MEG/EEG Group Study With the MNE Software: Recommendations, Quality Assessments, and Good Practices	Jas et al.	MNE site	BSD	EEG/ MEG	General purpose	Python	MNE	Yes (HW)
Analysis of Functional Connectivity and Oscillatory Power Using DICS: From Raw MEG Data to Group-Level Statistics in Python	van Vliet et al.	MNE site		EEG/ MEG	Connectivity analysis	Python	MNE	Yes (HW)
BrainWave: A MATLAB Toolbox for Beamformer Source Analysis of MEG Data	Jobst et al.	Brainwave site	GNU/ GPL	MEG	Beamformer	MATLAB		Yes
Bayesian Model Selection Maps for Group Studies Using M/EEG Data	Harris et al.	Sup. Mat		EEG/ MEG	Bayesian Model Selection Maps	MATLAB	SPM	Yes
Task-Evoked Dynamic Network Analysis Through Hidden Markov Modeling	Quinn et al.	GitHub		MEG	Dynamic Network Analysis using Hidden Markov Models	MATLAB	SPM; OSL	Yes (HW)
FieldTrip Made Easy: An Analysis Protocol for Group Analysis of the Auditory Steady State Brain Response in Time, Frequency, and Space	Popov et al.	Fieldtrip site	GNU/ GPL	EEG/ MEG	General purpose	MATLAB	Fieldtrip	Yes
Estimating the Timing of Cognitive Operations With MEG/EEG Latency Measures: A Primer, a Brief Tutorial, and an Implementation of Various Methods	Liesefeld	GitHub		EEG/ MEG	Timing of cognitive operations	MATLAB	Fieldtrip	Yes (HW)
MEG/EEG Group Analysis With Brainstorm	Tadel et al.	Brainstorm site	GNU/ GPL	EEG/ MEG	Group analysis; Source localization	MATLAB	Brainstorm	Yes (HW)
MEG Source Imaging and Group Analysis Using VBMEG	Takeda et al.	VBMEG site	GNU/ GPL	MEG	MRI based connectivity analysis	MATLAB	Freesurfer	Yes (HW)
Brainstorm Pipeline Analysis of Resting-State Data From the Open MEG Archive	Niso et al.	Brainstorm site	GNU/ GPL	MEG	Resting state analysis	MATLAB	Brainstorm	Yes
Multimodal Integration of M/EEG and f/MRI Data in SPM12	Henson, et al.	Figshare		EEG/ MEG/ fMRI	Multimodal integration of EEG/MEG with fMRI	MATLAB	SPM	Yes (HW)
NUTMEG: Open Source Software for M/EEG Source Reconstruction	Hinkley et al.	NUTMEG site	GNU/ GPL and BSD	EEG/ MEG	EEG/MEG source reconstruction	MATLAB	NUTMEG	Yes
From BIDS-Formatted EEG Data to Sensor-Space Group Results: A Fully Reproducible Workflow With EEGLAB and LIMO EEG	Pernet et al.	LIMO site	GNU/ GPL and BSD	EEG	Automated processing; EEG statistical analysis	MATLAB	EEGLAB and LIMO	Yes (HW)

*HW stands for Henson Wakeman face dataset. Sup. Mat. indicate that the article processing scripts are available in supplemental material.*

† *Data not referenced in the article but available at https://zenodo.org/record/998965*.

The articles in this special issue focus on different aspects of MEEG data processing. Some articles processed EEG data (*n* = 9), MEG data (*n* = 8), joint EEG/MEG data (*n* = 7), or even EEG/MEG/fMRI data (*n* = 1). Four articles focused on automated processing of EEG data, 10 dealt

with source localization, 3 with connectivity analysis, 3 with statistical analysis, 2 with EEG data classification. Other topics included microstates and Bayesian modeling. Submissions were based on existing MEEG software, in particular EEGLAB (*n* = 7), FieldTrip (*n* = 7), MNE (*n* = 4), SPM (*n* = 3), Brainstorm (*n* = 2), and NUTMEG (*n* = 1). Of the 25 articles, 21 are using MATLAB, 4 are using Python, and 1 is partially using R. Most scripts and tools were released under the GNU/GPL license (*n* = 10), BSD or MIT commercial friendly license (*n* = 2), no specific license (*n* = 11), or a combination of licenses (*n* = 2).

For researchers starting to process MEG/EEG data, we would recommend downloading the HW dataset (https://doi.org/10.18112/openneuro.ds000117.v1.0.5) and trying the methods described in this special issue. A simplified BIDS version of this dataset with EEG only is also available (https://doi.org/10.18112/openneuro.ds002718.v1.0.5). Furthermore, we recommend researchers to format their own data to BIDS to facilitate the application of some of the tools in this special issue and help the field move toward better tool integration centered on the BIDS framework.

Overall, there is tremendous potential in using different tools to process the same datasets. First, it forces tool developers to use a standard data format (BIDS) and increases interoperability between tools. Second, these tools offer common features, so the community may compare and check the numerical validity of each approach. Validity checking of MEEG signal processing approaches is important for open-source software, which often has limited resources assigned for testing purposes. Being able to process the same dataset using different tools also makes it simpler for users to compare them and see which one fits their style best, whether it is mixed GUI/script tools like EEGLAB, Brainstorm, SPM and NUTMEG or pure scripting tools such as Fieldtrip or MNE. Finally, making it possible to combine the signal processing pipelines of different tools allows users to develop approaches, leading to new methodological developments.

## Author Contributions

AD wrote the manuscript. RO, FT, AG, SN, and VL edited the manuscript. All authors contributed to the article and approved the submitted version.

## Conflict of Interest

The authors declare that the research was conducted in the absence of any commercial or financial relationships that could be construed as a potential conflict of interest.

## Publisher's Note

All claims expressed in this article are solely those of the authors and do not necessarily represent those of their affiliated organizations, or those of the publisher, the editors and the reviewers. Any product that may be evaluated in this article, or claim that may be made by its manufacturer, is not guaranteed or endorsed by the publisher.
